# Data storage using peptide sequences

**DOI:** 10.1038/s41467-021-24496-9

**Published:** 2021-07-13

**Authors:** Cheuk Chi A. Ng, Wai Man Tam, Haidi Yin, Qian Wu, Pui-Kin So, Melody Yee-Man Wong, Francis C. M. Lau, Zhong-Ping Yao

**Affiliations:** 1 State Key Laboratory of Chemical Biology and Drug Discovery, Research Institute for Future Food and Department of Applied Biology and Chemical Technology, The Hong Kong Polytechnic University, Hung Hom, Kowloon, Hong Kong SAR, China; 2 State Key Laboratory of Chinese Medicine and Molecular Pharmacology (Incubation) and Shenzhen Key Laboratory of Food Biological Safety Control, The Hong Kong Polytechnic University Shenzhen Research Institute, Shenzhen, China; 3 Department of Electronic and Information Engineering, The Hong Kong Polytechnic University, Hung Hom, Kowloon, Hong Kong SAR, China; 4 University Research Facility in Life Sciences, The Hong Kong Polytechnic University, Hung Hom, Kowloon, Hong Kong SAR, China; 5 University Research Facility in Chemical and Environmental Analysis, The Hong Kong Polytechnic University, Hung Hom, Kowloon, Hong Kong SAR, China

**Keywords:** Proteomics, Biotechnology, Computational biology and bioinformatics, Mass spectrometry

## Abstract

Humankind is generating digital data at an exponential rate. These data are typically stored using electronic, magnetic or optical devices, which require large physical spaces and cannot last for a very long time. Here we report the use of peptide sequences for data storage, which can be durable and of high storage density. With the selection of suitable constitutive amino acids, designs of address codes and error-correction schemes to protect the order and integrity of the stored data, optimization of the analytical protocol and development of a software to effectively recover peptide sequences from the tandem mass spectra, we demonstrated the feasibility of this method by successfully storing and retrieving a text file and the music file Silent Night with 40 and 511 18-mer peptides respectively. This method for the first time links data storage with the peptide synthesis industry and proteomics techniques, and is expected to stimulate the development of relevant fields.

## Abstract (web summary)

Finding durable, high-density media for data storage is necessary to support the ever-expanding generation of digital data. Here, the authors use peptide sequences to store digital data and retrieve them using tandem mass spectrometry, proving that peptides can be used as a storage medium.

## Introduction

From the beginning of civilization, the media for storing data have been continuously evolving from such as stone tablets, animal bones, and bamboo tablets to paper, with improvements on data density over time. Since the invention of electronics in the last century, the percentage of data stored in digital form has been increasing rapidly to almost 100% recently^1^. Moreover, the amount of data generated has been increasing exponentially, from several ZB in 2008 to expected 74 ZB in 2021, causing a much increased demand for data storage correspondingly^2^. Most of the digital data are stored in physical media such as hard drives. In addition, many of the data are rarely accessed and are archived on reels of magnetic tapes. However, the physical thickness of the tapes and the size of magnetic domains limit the maximum data density, which is expected to reach a plateau soon. Furthermore, data in old tapes need to be copied onto new tapes regularly, as the magnetic tapes can normally last for 10 to 20 years only. This process is time-consuming and expensive. Hence, next-generation media that can store digital data with a much higher data density and durability are needed.

One of the emerging technologies to fulfill this need is storing digital data in molecules. A widely reported technique is data storage with deoxyribonucleic acid (DNA), where the capability of DNA data storage had advanced from several bytes decades ago^3^ to hundreds-MB-scale recently^4–6^. While early examples did not achieve complete data recovery^7^, the data integrity has been improving by incorporating error-correction schemes in DNA data storage, from simple repetitions^8^ towards more complex and efficient schemes such as Reed–Solomon (RS) code^9^ and fountain code^10^. DNA could offer much higher data density than magnetic tapes^11^ and store information for thousands of years^9^.

In addition to DNA, other molecules were investigated for storing digital data. For example, Roy et al. encoded data in synthetic polymers such as poly(alkoxyamine amide)s^12^, and Huang et al. transformed the data into binary trees and encoded the transformed data in dendrimers^13^. Very recently, Cafferty et al. encoded data in organic molecules using a set of molecules with different masses representing the 0 and 1 in digital data and read out using matrix-assisted laser desorption/ionization mass spectrometry^14^.

Here we report the use of peptide sequences for digital data storage, a method that has not been reported before^15^. Compared to DNA and other types of polymers, peptides offer several advantages for data storage. Firstly, in DNA, typically only four natural nucleotides are used as monomers due to the requirement of enzyme recognition for PCR amplification and high-throughput sequencing. In synthetic peptides, a much greater variety of monomers (amino acids) can be incorporated because enzyme recognition is not mandatory in the synthesis and sequencing of peptides. In addition to the 20 natural amino acids, many unnatural amino acids can be used. The increased set of possible monomers and lower masses than those of nucleotides could in principle allow peptides to have a higher density than DNA for data storage. Secondly, peptides can be more stable than DNA. It has been shown that after millions of years, peptides or proteins could still be detected and sequenced but DNA had already degraded^16,17^. Comparing to DNA, peptides cannot be amplified with techniques such as polymerase chain reaction (PCR). However, using tandem mass spectrometry (MS/MS)-based techniques, peptides can be detected and sequenced with good sensitivity and direct data readout without PCR-like preprocessing^18–20^. Moreover, the field of proteomics has been developing rapidly with constantly improving methods, hardware and software to allow sequencing of thousands of peptides within a very short time;^21,22^ the peptide synthesis industry has been established, and the price for peptide synthesis continues to decrease. Thus comparing to other polymers or small molecules, peptides could better leverage the established methodologies and industry for design and sequencing.

## Results and discussion

**Fig. 1:**
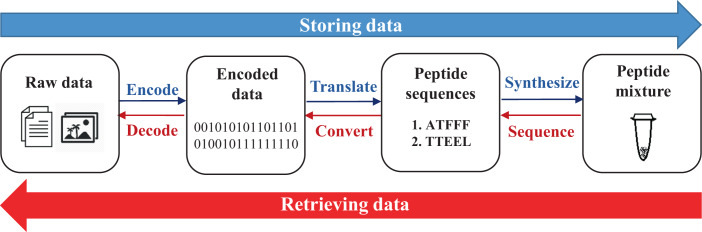
Overview of the process of storing and retrieving data into and from peptides. The direction in blue represents the data storing process, while the direction in red represents the data retrieving process.

We have developed a method for data storage using peptide sequences, with the precise ordering of amino acids encoding the order of digital bits. As shown in Fig. 1, in our method, amino acids are assigned as sequences of digital bits (Table 1). Raw data are first encoded as long strings of 0 s and 1 s, which correspond to sequences of amino acids, i.e., peptides, according to the assignments. The peptides are synthesized and hence the data are stored. To retrieve the data, the peptides are sequenced, and the obtained sequences are converted into bits of 0 and 1, which are then decoded as the raw data. The peptides can be commercially synthesized, and MS/MS is the state-of-the-art technique for peptide sequencing. Peptides must not be too long in order to ensure effective synthesis and sequencing. Therefore, the encoded strings are broken into smaller parts, and an address indicator is added into each part to ensure all the parts will be in their original order when they are read back. In this way, raw data will be stored in a mixture of peptides, which can be separated and sequenced using liquid chromatography coupled with MS/MS (LC-MS/MS). The keys of this method are the successful synthesis, detection and sequencing of all the peptides, which have been achieved by selecting suitable amino acids to comprise the peptides, designing suitable error-correction coding schemes, optimizing the protocol for LC-MS/MS analysis, and developing a software to effectively recover peptide sequences from the MS/MS spectra. These efforts are illustrated below, with more details available in Methods and Supplementary Information sections.

**Table 1:** The one-to-one mapping of bit sequences to amino acids.

In proteomics studies, thousands of proteins could be reliably identified in one LC-MS/MS analysis, even with low sequencing coverage of the peptides, since the peptides are originated from proteins with sequences available in databases for searching^21,22^. However, such a strategy cannot be used for sequencing data-bearing peptides, which requires nearly all the amino acids of each peptide to be correctly sequenced in order to recover all encoded information. De novo peptide sequencing, a technique based on high accuracy MS/MS^23,24^ and widely used for sequencing of monoclonal antibodies in industry^25^, was thus used to sequence the encoded peptides. Fortunately, different from proteomic peptides with totally random and unknown sequences, the peptide sequences used for data storage can be designed beforehand according to some rules such that the sequencing accuracy is optimized.

For the peptide design, we considered several parameters with an aim to increasing the success rate of complete sequencing. The first parameter is the peptide length. Shorter peptides are easier to be synthesized and sequenced with fewer missed fragmentation, while longer peptides could store more data per peptide, reducing the number of peptides required as well as the number of addresses and error correction overhead for the same amount of data. To balance these factors, the peptide length was fixed to 18-mer long in this study. The second parameter is the choice and positioning of amino acids. Among the 20 natural amino acids, proline (P) was eliminated as peptides containing P are difficult to synthesize^26^. Histidine (H), lysine (K), and arginine (R) were not used in the middle or at the N-terminus as they caused sharp decrease in peak intensity^27,28^. Methionine (M) and cysteine (C) were eliminated because they were prone to oxidization and formation of disulfide bridges, respectively. Asparagine (N) and glutamine (Q) were eliminated as they were prone to amine loss during fragmentation in MS/MS^27^. Isoleucine (I) was eliminated as it is isobaric with leucine (L). From the 11 remaining amino acids, eight amino acids, i.e., alanine (A), valine (V), leucine (L), serine (S), threonine (T), phenylalanine (F), tyrosine (Y) and glutamic acid (E), were selected to comprise the data storage peptides with 3 bits per amino acid (Table 1). (Note that it requires 16 amino acids in order to encode 4 bits per amino acid.) C-terminal arginine has been found to promote the signal intensity of the y-ion series and suppress the b-ion series^28^. As this would simplify spectral analysis, a non-data-bearing amino acid, R, was placed at the C-terminus for each peptide. As the first and second amino acids from the N-terminus were rarely fragmented in MS/MS^29^, another non-data-bearing amino acid, F, was fixed as the first amino acid from the N-terminus, so that the mass of the second amino acid could still be calculated when the first and second amino acids failed to be fragmented. Another reason for fixing F at the N-terminus was to balance the hydrophobicity of peptides, as F was hydrophobic, while R that was fixed at the C-terminus was hydrophilic. Peptides with medium hydrophobicity could facilitate peptide synthesis, as the solubility of hydrophobic peptides is low, and very hydrophilic peptides are difficult to be purified by HPLC. These choices would produce peptides that could be easily synthesized and chemically stable. They could also generate MS/MS spectra that easily allow correct peptide sequence recovery.

To further protect data integrity, error-correction schemes^30^ were incorporated during encoding, such that when peptides were not synthesized, detected, or sequenced well, the missing data could still be inferred from the appended redundant data^9,10^. In this study, we designed a concatenated error-correction code, assuming that 10% of amino acids were missing or incorrect during storage and retrieval, and the orders of the second and third amino acids counted from both N-terminus (Table 2, symbols S_1_ and S_2_) and C-terminus (Table 2, symbols S_15_ and S_16_) might be ambiguous because gap masses due to fragmentation were more common on these sites. The error of 10% missing amino acids was protected using an advanced low-density parity-check (LDPC)^31^ or RS^32^ code, while the error of ambiguous order of specific amino acids was protected by two bits, with each bit protecting the order of the second and third amino acids on each end of the peptide. From the design of the peptide structure, each amino acid represented one symbol, which contains 3 bits of information (Table 1, S1 and S2).

**Table 2:** Structure of sequences with 16 3-bit symbols, where each 3-bit symbol can be translated to one amino acid according to Table 1.

The first 2 or 3 symbols are used to assign the address (“Add”, light red). The bit *Q*_*i,j*_ is used to record the order of *S*_*i*_ and *S*_*j*_ (white). The other symbols are used to store (i) the coded bits *c* (green) including the information and the parity bits; and (ii) some zero bits (blue) to ensure that at least three symbols are hydrophilic amino acids.

**Fig. 2:**
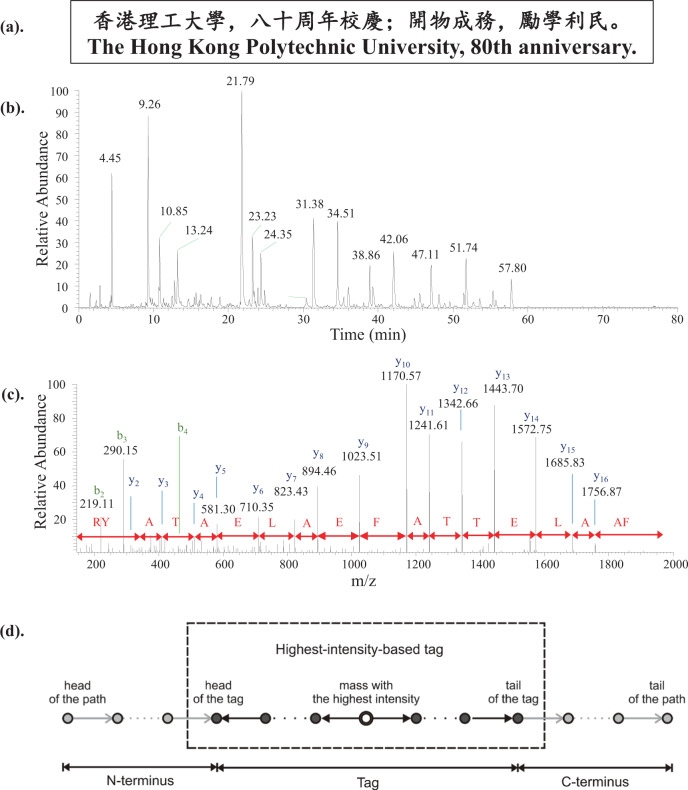
Overview of data retrieval from dataset A. **a** The message of dataset A; **b** The chromatogram for analysis of the 40 peptides for dataset A; **c** A typical MS/MS spectrum for analysis of peptides in dataset A, and the sequence of one of the data-bearing peptide read out from the spectrum; **d** The highest-intensity-tag-based sequencing method used in the sequence recovery.

To retrieve the data from the stored peptides, the peptide mixtures were separated with LC and then subjected to fragmentation to produce MS/MS spectra that allowed recovery of the amino acid order based on the mass differences between the fragment ions (Fig. 2b, c). Currently available proteomics and de novo sequencing software^21–25,29^ were found not to work well for the sequence recovery since they were not developed for the specific peptides used in this project. An in-house software using a highest-intensity-tag-based method (Fig. 2d), tailored to the arrangements of amino acids of the designed peptides, was thus developed to recover peptide sequences from the MS/MS spectra (Table S3) in this project. The peptide sequences were then grouped by scoring and finally decoded to recover the original data.

**Fig. 3:**
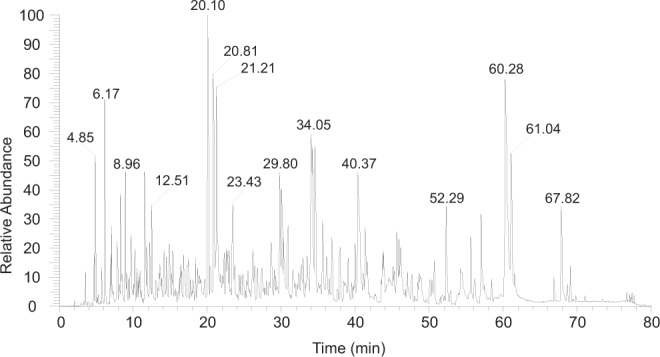
The chromatogram for analysis of the peptide mixture encoding dataset B.

As proofs of concept, two datasets were stored and retrieved in this study. Dataset A was an 848 bits long BIG5-formatted text for “The Hong Kong Polytechnic University, 80th anniversary.” in both Chinese and English and the motto of The Hong Kong Polytechnic University in Chinese (Fig. 2a), while dataset B was 13,752 bits long, containing the music Silent Night in MIDI (Supplementary Audio 1) format and its title in ASCII format. Dataset A was encoded (Table 1 and S1) and translated into 40 18-mer peptides (Table S4), which were synthesized for data storage. For data retrieval, the peptide mixture was analyzed using LC-MS/MS (Fig. 2b), and the acquired MS/MS spectra (Fig. 2c) were processed with the in-house software for recovery of the peptide sequences, which were converted back to sequences of bits according to the previous assignments (Table 1) and then decoded back to the original raw data. The results showed that the sequences of all 40 peptides were correctly obtained, allowing complete retrieval of the original data. Similar procedures (Fig. 3 and Table 1 and S2) were employed for storage and retrieval of dataset B, which required 511 18-mer peptides for data storage. The results showed that 93.7% (7659/8176) of the amino acids were correctly recovered (Table S5). After the error-correction decoding procedure that could recover a maximum of 10% of incorrect or lost amino acids, the original music and title were fully retrieved.

In DNA data storage that used four nucleotides as monomers, each nucleotide represented 2 bits, while the use of eight amino acids as monomers in our method enabled each amino acid to represent 3 bits. Together with the lower masses of amino acids, in principle, the storage density of our method could be 3.72 times of the DNA method, i.e., storage of the same data using a lower amount (lighter) of peptides than DNA. The storage density of our method can still be further improved with use of 16 or more amino acids. Practically, the retrievable data density was 1.7 × 10^10^ bits/g and 2.6 × 10^9^ bits/g for datasets A and B, respectively, which were about nine orders of magnitudes lower than those of the DNA method^11^. The major reason for this is that DNA can be amplified by PCR prior to sequencing while peptides cannot, therefore the number of molecules required to retrieve data for the DNA method can be far fewer than the peptide method. The peptide-based data density can be significantly improved with optimized peptide sequencing, since picomole amounts of peptides were used for analysis in these proof-of-concept studies while peptide detection and sequencing at attomole^18–20^, yoctomole^33^, or even single molecule^34–36^ scales have been reported.

In summary, we demonstrated that it was feasible to store data using peptide sequences and to retrieve the data using LC-MS/MS analysis. This method offers a new possibility for data storage with potentially high storage density and durability. Peptide synthesis industry and proteomics techniques have been developed to the stages that can allow the use of peptides for data storage. Our method for the first time connects these fields together and can promote the development of these and other relevant fields. Currently, peptide synthesis and sequencing are still relatively expensive and time-consuming in practice, and scaling-up significantly would require further developments in these fields. As the stored data become much larger, much more peptides would be required to encode the data, leading to much more complicated peptide sequencing that would challenge the analytical capabilities of current LC-MS/MS techniques, and new analytical techniques and strategies would be needed to solve the problems. However, with the improved techniques and reduced time and costs of the peptide synthesis and sequencing, which have been happening in the past decades, peptide data storage may become practically available in the future, especially in critical applications that demands minimum weight and long duration for stable storage of very big data.

## Methods

### Materials

Peptides (lyophilized, as trifluoroacetate salts, >50% purity) were synthesized by Genscript Inc. (Nanjing, China) and GL Biochem (Shanghai, China). The peptides were dissolved in dimethyl sulfoxide (10 µg/mL), mixed together for each dataset, and diluted with 50% acetonitrile with a 1:1 ratio before analysis. Methanol and acetonitrile (HPLC grade) were from Duksan (South Korea). Formic acid (99–100%) was from VWR (France). Water was purified by MilliQ system.

### LC-MS/MS analysis of the peptide mixtures

The step-by-step protocol used in this work is available on Protocol Exchange^37^. The peptide mixtures were separated using a Waters Acquity UPLC system with a C18 column (Agilent AdvanceBio Peptide Map, 2.1 × 150 mm, 2.7 µm particle size, 120 Å pore size). Mobile phase A was 0.2% formic acid in water and B was 0.2% formic acid in acetonitrile. The flow rate was 0.3 mL/min and the temperature was 55 °C. The gradient changed from 10% B to 18% B at 0 to 2 min, from 18% B to 22% B at 2 to 8 min, from 22% B to 34% B at 8 to 48 min, from 34% B to 40% B at 48 to 64 min, from 40% B to 55% B at 64 to 75 min, from 55% B to 80% B at 75 to 78 min, and remained at 80% B from 78 to 83 min.

MS/MS analysis was performed using an Orbitrap Fusion Lumos mass spectrometer (ThermoFisher Scientific, San Jose, CA) operated in positive ion mode. The spray voltage for electrospray ionization was +3600 V, and both ion transfer tube temperature and vaporizer temperature were 280 °C. In each cycle, a MS1 scan with *m/z* from 900 to 1400 Da was performed with a resolution of 30 K. Ions were selected for MS/MS with quadruple, using advanced peak determination (APD) with default charge of +2, top-speed mode with 3 s cycles, mass tolerance of 25 ppm, dynamic exclusion window of 4 s, and isolation window width of 1.6 or 0.7 Da. High-energy collision dissociation (HCD) at 28% of normalized collision energy with stepped collision energy of 5% was used for the fragmentation. MS/MS spectra were obtained with *m/z* from 240 to 2450 Da and a resolution of 15 K.

### Error-correction code design

The structures of the sequences used for encoding, sequencing, and decoding are shown in Table 2 for datasets A and B.

For dataset A, the first two symbols *S*_1_ and *S*_2_ are used to assign the address (orange). The order-checking bits *Q*_1,2_ and *Q*_15,16_ are used to record the order of *S*_1_ and *S*_2_ and the order of *S*_15_ and *S*_16_, respectively (white). Note that 3 zero bits (blue) are filled in the first bits of *S*_4_–*S*_6,_ which can ensure that at least three symbols are hydrophilic amino acids. The other symbols are used to store the coded bits *c* including the information and the parity bits (green) of the error-correction codes. For dataset B, due to more information bits and longer address, three address symbols and three order-checking bits are used. Such structure can be easily modified for longer sequence with longer address.

As shown in Table S4, a 40 × 16 block is constructed for error correction and peptide sequencing of dataset A. Each row had 16 symbols (i.e., *S*_1_, *S*_2_, …, *S*_16_) to represent a 16-mer data-bearing peptide sequence. In the design of encoding scheme for dataset A, the first two symbols in each sequence were used to store the address, and the remaining 14 symbols were used to store information. Hence a total of 560 symbols are available in the 40 peptide sequences (total 560 × 3 = 1680 bits, see Table S2 for the 40 peptide sequences). Then 850 information bits (i.e., *b*_1_, *b*_2_, …, *b*_850_) were filled in the data block according to the following arrangements (Table S4):

- Bits *b*_1_–*b*_400_ were filled in the second and the third bits of Symbols *S*_3_–*S*_7_ of the peptide sequences Seq #1 to Seq #40;
- Bits *b*_401_–*b*_760_ were filled in Symbols *S*_14_–*S*_16_ of the peptide sequences Seq #1 to Seq #40;
- Bits *b*_761_–*b*_850_ were filled in Symbol *S*_13_ of the peptide sequences Seq #11 to Seq #40.

Furthermore, the first bits of Symbols *S*_4_–*S*_6_ (represented by *b*_851_–*b*_970_) were filled with “0” bits. The purpose is to ensure that a minimum of three symbols in each sequence having values 0, 1, 2, 3, which will be represented by hydrophilic amino acids S, T, E, Y, as mentioned in the design. There were also order-checking bits *Q* and redundant bits *P*_*i*_^*(j)*^ (*i* = 1, 2, …, *n*; *j* = 1, 2, and 3) derived by LDPC codes in the peptide sequences (where *n* is the number of parity bits in each LDPC code). The order-checking bit *Q*_*i,j*_ is “1” if symbol *S*_*i*_ is larger than *S*_*j*_. Otherwise, the order-checking bit is “0”. Thus, the maximum overall code rate *R* of the block was 850/(14 × 3 × 40) = 0.506. As dataset A only consisted of 848 bits, 2 zeros were appended to the end of the data in order to fully fill the block.

Based on the results of dataset A, we further made these assumptions of possible errors when designing the error-correction scheme for dataset B: (i) 10% of the three-symbol sequences {*S*_5_*S*_6_*S*_7_} and {*S*_8_*S*_9_*S*_10_} cannot be recovered correctly; and (ii) 15% of the three-symbol sequences {*S*_11_*S*_12_*S*_13_} and {*S*_14_*S*_15_*S*_16_} cannot be recovered correctly. Based on these assumptions, we proposed another error-correction method based on the RS code^32^ that used: (i) three order-checking bits for each peptide sequence; and four RS codes to recover the original data even when any arbitrary 10% three-symbol sequences {*S*_5_*S*_6_*S*_7_}, any arbitrary 10% three-symbol sequences {*S*_8_*S*_9_*S*_10_}, any arbitrary 15% three-symbol sequences {*S*_11_*S*_12_*S*_13_}, and any arbitrary 15% three-symbol sequences {*S*_14_*S*_15_*S*_16_}, cannot be recovered correctly.

When this scheme was used on dataset B, a 511 × 16 block of symbols was constructed (Table S5), which comprises 511 × 16 × 3 = 24528 bits. The three-symbol sets *A*_*i*,1_ *A*_*i*,2_ *A*_*i*,3_ (*i* = 1, 2, …, 511) were used for addressing, with Symbols *S*_1_ to *S*_3_ having the values of 000, 001, 002, …, 775, 776. The three bits of Symbol *S*_4_ were the three order-checking bits used to protect the order of Symbols *S*_1_ and *S*_2_, the order of Symbols *S*_2_ and *S*_3_, and the order of Symbols *S*_15_ and *S*_16_, respectively. Then there were 511 × 12 × 3 = 18396 bit positions in Symbols *S*_5_ to *S*_16_ of the block to store the information and parity bits for the RS codes. Due to the different protection requirements for the partial sequences {*S*_5_*S*_6_*S*_7_*S*_8_*S*_9_*S*_10_} and {*S*_11_*S*_12_*S*_13_*S*_14_*S*_15_*S*_16_}, two (511, 409) RS codes were used for partial sequences {*S*_5_*S*_6_*S*_7_} (RS1) and {*S*_8_*S*_9_*S*_10_} (RS2), another two (511, 357) RS codes were used for partial sequences {*S*_11_*S*_12_*S*_13_} (RS3) and {*S*_14_*S*_15_*S*_16_} (RS4). Each symbol in RS code comprised 9 bits. The (511, 409) RS and (511, 357) RS codes could correct up to 51 and 77 9-bit symbol errors, respectively (Table S5). Moreover, the numbers of total information bits and total parity bits of all four RS codes were given by (409 + 357) × 2 × 9 = 13788 and (102 + 154) × 2 × 9 = 4608, respectively. The maximum overall code rate *R* of the block is given by 13788/(511 × 16 × 3) = 0.562. As dataset B only had 13752 bits, zeros were appended to the end such that the block could be fully filled. This code rate is comparable to that of DNA (varied from 0.17 for repetition encoding^8^ to 0.785 for fountain encoding^10^, assuming a maximum capacity of 2 bits per nucleotide). Improvement of code rate is possible if longer peptides could be used, if the peptide design could be improved to reduce error, and if the coding scheme could be more focused on the error-prone amino acid positions to reduce unnecessary redundancy.

### Sequence recovery

An in-house software was developed for recovery of peptide sequences from the MS/MS spectra. To reduce the amount of false positives, during spectral analysis, the maximum error for each peak was set to 25 ppm (in line with the experimental parameters), and the masses were corrected to at least five decimal places.

The spectra were first extracted from the Thermos RAW file using MSConvert^38^. Then, the spectra were passed onto a preprocessing unit, which included deconvolution to obtain a list of masses and charges of isotopic clusters with one or more peaks each, and identifying the monoisotopic mass and charge of parent ion. As only 2+ precursor ions were selected for MS/MS during experiments, most fragments would be predominately 1+ unless their masses were close to precursor mass. Also, it was predicted that in most peptides, the strongest peak in the isotopic cluster would be M + 1 rather than M if the formula mass were above ca. 1800^39^, where M was the monoisotopic mass. Therefore, it was assumed that if a certain isotopic cluster only consisted of one peak, that peak was singly charged and the monoisotopic mass would be corrected based on the deconvoluted mass accordingly. MS/MS spectra with less than 12 peaks with the most intense peak lower than 30,000 counts were filtered out at this stage to speed up analysis.

**Fig. 4:**
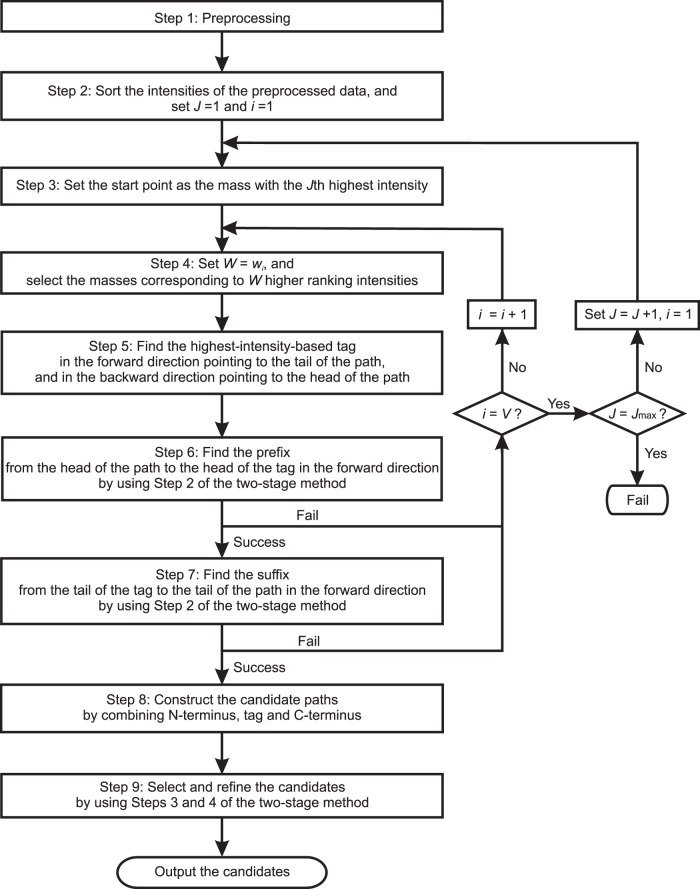
A flowchart illustrating the method of highest-intensity-tag based sequencing. *i* represents the iteration number and *V* is the maximum number of iterations. *W* represents the number of masses with the higher ranking used in the tag-finding processing and *w*_*i*_ is the number of masses with the higher ranking for the *i*th iteration. *J* represents the ranking of intensity and *J*_max_ is the maximum number of higher-ranking-intensity masses allowed to be the start point to find the tag.

After that, de novo sequencing based on the graph model is utilized for determining the peptide sequences from the preprocessed spectra^23^. In the graph model, the MS/MS spectrum is represented by a directed acyclic graph (DAG). The peaks of the spectrum can be taken as vertices, while an edge is added between two vertices when the mass gap between two peaks is equal to the mass of an amino acid. The objective is to find the longest path in the graph starting from the head vertex to the tail vertex. The sequence identification was started in the middle part of the MS/MS spectrum. The sequence tagging method first infers a partial sequence called tag, and then finds the whole sequence that can match the tag. The tags containing the amino acid with the highest intensity were first obtained, which were called highest-intensity-based tags (Fig. 4). To generate valid sequence candidates, both ends of the tag would be extended and connected to the N- and C- termini by searching the sequences containing amino acids with matching gap masses. The scores for the sequence candidates based on the following five factors: the length of consecutive amino acids retrieved, the number of amino acids retrieved, match error, intensity, and the number of occurrences for different ion types with different offsets. The higher the score is, the more likely that the sequence is correct.

### Details of the highest-intensity-tag based sequencing method

In the highest-intensity-tag based sequencing method (Fig. 4), the sequences were estimated in a manner that first inferred a partial sequence with a small amount of reliable information and then found the missing part of the sequence with less reliable data or the raw data. The *m/z* value with the first highest intensity was first recognized to further infer the tag or the path. Although using short tags (e.g., with three amino acids) such as GutenTag^40^, DirecTag^41^, and NovoHCD^42^ could avoid introducing the wrong amino acids, the number of candidate tags would be relatively larger and harder to infer the sequences due to insufficient information provided by the tag. Therefore, in this project, the length of tag was variable and could be up to the length of the peptide, which helped to reduce the search space. When a tag contained wrong amino acids, it could not be extended well towards N-terminus and C-terminus. In this case, the length of the tag was shortened by adaptively reducing the number of the higher-intensity data points used for the tag-finding algorithm. In addition, the vertex with the highest intensity may not definitely present in the correct path due to the uncertainty of the data. When valid paths could not be found, it may be possible to infer the tag with the second or even the third highest intensities. Moreover, in order to find the tag, N-terminal and C-terminal amino acids, a sequencing method called two-stage sequencing method is used together with the highest-intensity-tag based method.

Figure 4 shows a flowchart illustrating the method of highest-intensity-tag based sequencing. At steps 2, 3 and 4, the intensities of the preprocessed data were sorted from the largest to the smallest and values with *J* denoting the ranking of intensity. The mass/charge ratio with the highest intensity was then identified. At start, it was set as *J* = 1 and *i* = 1, and using only *W* = *w*_*i*_ (*w*_1_ > *w*_2_ > *w*_3_*…*) masses with the higher ranking in the tag-finding processing.

The method then proceeded to step 5 to find the highest-intensity-based tag. Starting from the mass of the putative y-ion with the highest intensity, the highest-intensity-based tag was found by simultaneously connecting the vertices in the forward direction pointing to the tail vertex of the path, and connecting the vertices in the backward direction pointing to the head vertex of the path, where the vertices had mass gap being the exact mass of any amino acid and preferably the length of the tag was as long as possible (Fig. 2d). The tags containing the amino acid with the highest intensity were obtained subsequently, which were called highest-intensity-based tags. With knowledge of the masses of the head and the tail amino acids of a highest-intensity-based tag, the method proceeded to step 6 to find the N-terminal amino acids that could connect the head of the path to the head of the tags in the forward direction by using the method described at Step 2 of the two-stage sequencing method. Similarly, for the tags with valid N-terminal amino acids, at step 7, the C-terminal amino acids of the sequences could be further found by connecting the tail of the tags to the tail of the path in the forward direction.

At step 8, the candidate paths could be constructed by combining the three parts: N-terminus, tag, and C-terminus. At step 9, one could follow steps 3 and 4 of the two-stage sequencing method to select and refine the sequences. Note that a larger value for *W* sometimes introduced one or more wrong amino acids in the head and/or tail parts of a tag, while a smaller value for *W* may give more reliable tag but the length of the tag may be limited. Therefore, after step 6, if no valid candidate could be found, one may attempt to reduce the value of *W* with *W* = *w*_*i*_ by increasing *i* by 1, i.e., *i* = *i* + 1, and repeat the tag, N-terminus and C-terminus finding procedure until the candidate sequence could be found or *i* = *V* (where *V* is the maximum number of iterations).

For the special case when the experimental mass with the highest intensity gave an unreliable message due to noise and uncertainty, a highest-intensity-based tag or a valid path with the highest-intensity-based tag could not be found. In this case, the mass with the second highest intensity was used by setting *J* = *J* + 1 and *i* = 1 to find the second highest-intensity-based tag and the candidates. This process would continue until the sequence could be found or *J* = *J*_max_ (where *J*_max_ is the maximum number of higher-ranking-intensity masses allowed to be the start point to find the tag).

### Details of the two-stage sequencing method

**Fig. 5:**
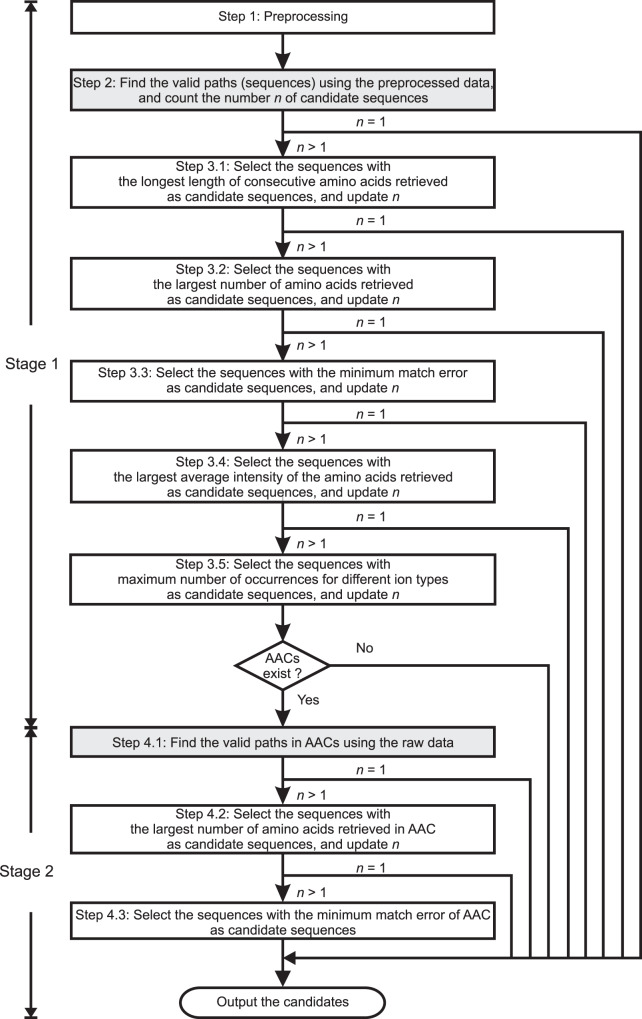
A flowchart illustrating the method of two-stage sequencing. AAC stands for amino acid combinations.

Figure 5 shows a flowchart illustrating a method of two-stage sequencing. Four steps are involved in the two-stage sequencing method: (1) preprocessing, (2) candidate sequence generation, (3) sequence selection, and (4) candidate refining. As shown in Fig. 5, Steps 1–3 belong to the first stage (Stage 1), while Step 4 is processed in the second stage (Stage 2). In Stage 1 of the two-stage sequencing method, partial sequence is inferred using the preprocessed data after Step 1. In Stage 2, the remaining part of the sequence is determined using the raw data.

At Step 1, preprocessing is performed. At Step 2, the preprocessed data from step 1 is used to find the valid paths (sequences), and the number *n* of candidate sequences is counted. At Step 3, the effects of the following five factors are jointly considered when arriving at the score of a sequence candidate from Step 3.1 to Step 3.5: length of consecutive amino acids retrieved, number of amino acids retrieved, match error, average intensity of amino acids retrieved, and number of occurrences for different ion types with different offsets. The sequences with the longest length of consecutive amino acids retrieved are first selected (Step 3.1). Among the selected sequences, the sequences with the largest number of amino acids retrieved are then selected (Step 3.2). For the sequences with equal length of consecutive amino acids retrieved together with equal number of amino acids retrieved, the match error is evaluated, which is the mean error between the observed mass values for the amino acids retrieved from the experimental spectrum and the actual mass values of the amino acids normalized by the corresponding observed mass values (Step 3.3). If there is more than one sequence with identical match errors, the average intensity of amino acids retrieved is further calculated and a higher score is given to a sequence with a larger average intensity value (Step 3.4). In addition, multiple ion types are usually considered as the important factors in inferring an amino acid, which means that a mass value may correspond to different types of ions in the spectrum. Generally, the more the number of occurrences for different ion types of an amino acid is, the more likely the amino acid is correct. Therefore, for the sequences with equal score after the aforementioned evaluations of Steps 3.1–3.4, the number of occurrences for different ion types is counted to determine the sequence (Step 3.5). The mass offset sets for the N-terminal a-ion, b-ion, and c-ion type sets, i.e., {a, a-H_2_O, a-NH_3_, a-NH_3_-H_2_O}, {b, b-H_2_O, b-H_2_O-H_2_O, b-NH_3_, b-NH_3_-H_2_O}, and {c, c-H_2_O, c-H_2_O-H_2_O, c-NH_3_, c-NH_3_-H_2_O} are {−27, −45, −44, −62}, {+1, −17, −35, −16, −34}, and {+18, 0, −18, +1, −17}, respectively. According to the fragmentation method and the property of the data, all or some of the above ion types can be used flexibly.

Since the candidate sequences obtained at Step 2 are found by using the preprocessed data, which aim to provide more reliable information to generate the partial sequence, amino acid combinations (AACs) may present in the sequence due to insufficient data provided by preprocessing. At Step 4, if selected sequences with missing mass values exist, which means that the corresponding mass gaps are equal to the summation of at least two amino acids, the raw data may be used to find as many vertices as possible for the path in Stage 2. After finding the missing amino acids of AACs at Step 4.1, the sequences with the longest length of consecutive amino acids retrieved in AACs are selected as candidate sequences (Step 4.2). If there still remain at least two candidate sequences after selection, a final decision is made based on the match error of the amino acids retrieved in AACs for each sequence (Step 4.3).

### Grouping

After sequence recovery, there could be more than one valid 18-mer sequences from each spectrum or multiple valid 18-mer sequences containing the same address. Therefore, sequencing selection and grouping were performed to identify the correct peptide for each address.

**Fig. 6:**
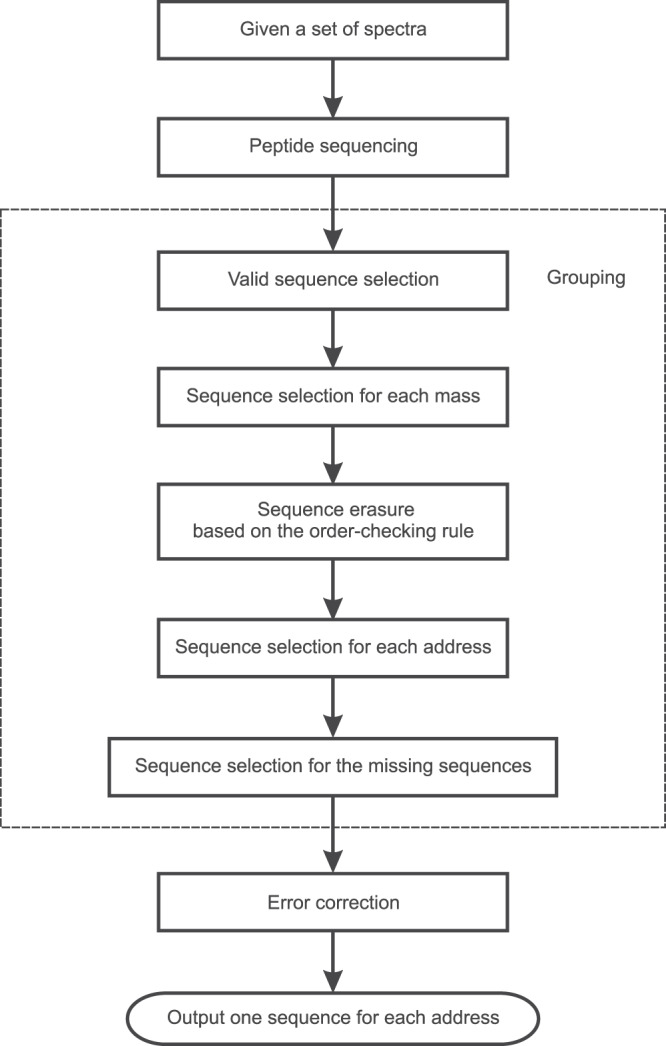
A flowchart illustrating the method of sequence grouping. The procedure of grouping is shown in the dashed square.

Given a set of spectra containing Ns (Ns = 40 or 511) peptide sequences. After sequencing, a set of sequence is obtained and a block of Ns × 16 is constructed for decoding. This process is called sequence grouping which is described below and in Fig. 6.

#### Valid sequence selection

To reduce the effect of the unreliable sequences caused by the noise and the uncertainty, the requirements for the valid sequence are listed as follows.

1. The sequence is of length-16.
2. For each sequence, one AAC with more than two missing amino acids is not allowed.
3. For each sequence, more than one AACs with two missing amino acids are not allowed.

After MS/MS analysis, a set of spectra containing 40 or 511 sequences is obtained, among which some spectra can generate length-16 sequences. If there are more than two missing amino acids in one AAC or two missing amino acids in more than one AACs, then the corresponding sequence will be ignored in the further selection.

#### Selection for each mass

For each mass value, if there are more than one output sequences with the highest score, then all these sequences are selected; otherwise, at most *L*_max_ (=2) sequences with higher scores will be considered for each of the selected spectra.

#### Erasure based on order checking

Based on the orders of the estimated symbol pairs {*S*_1_, *S*_2_} and {*S*_15_, *S*_16_} for 40 sequence set, 2 bits are generated according to the order-checking rule, which will be compared with the first bits of the estimated symbols *S*_3_ and *S*_7_, respectively. Similarly, 3 bits are generated based on the orders of the estimated symbol pairs {*S*_1_, *S*_2_}, {*S*_2_, *S*_3_}, and {*S*_15_, *S*_16_} for 511 sequence set, which will be compared with the 3 bits of the estimated symbol *S*_4_. If any one of the generated order-checking bits does not match the corresponding bit in an estimated sequence, the estimated sequence will be erased.

#### Selection for each address

According to the address represented by the first two and three elements of a sequence, the sequences are divided into 40 and 511 address groups, respectively. Then for each group, there are following cases possible:

Case 1: There is only one sequence.

Case 2: There are two or more sequences, some of which are the same, where:

2a. there is only one result with two or more sequences; or

2b. there are at least two different results, each with two or more sequences.

Case 3: All sequences in the group are different, where:

3a. different sequences belong to the same spectrum; or

3b. different sequences belong to different spectra.

For Case 1, the only sequence is recovered for the group. For Case 2a, the result with two or more sequences is selected. For Case 2b, the results with the largest number of sequences are first selected. Among the sequences corresponding to these results, the sequence with the highest score according to Steps 3.1–3.5, 4.2, and 4.3 of the two-stage sequencing method is further selected. For Case 3a, the sequence with the highest score according to Steps 4.2 and 4.3 of the two-stage sequencing method is selected. For Case 3b, the sequence with the highest score according to Steps 3.1–3.5, 4.2, and 4.3 of the two-stage sequencing method is selected.

#### Selection for missing sequences in the block

With knowledge of the sequences corresponding to each address, a *N*_*s*_ x 16 block of symbols can be constructed with each row of the block representing a sequence and each symbol representing an amino acid. In this block, some rows of the block may be missing due to the erasure by the order-checking process or the impurity of the data for peptide sequencing.

If there existed missing rows for some addresses in the block, the length-16 sequences generated by all spectra were considered to find these missing rows. For each address with missing row, the scores of the sequences were compared to make the decision.

### Calculation of data density

In theory, each nucleotide in DNA could hold 2 bits while each amino acid in our designed peptides could hold 3 bits. The average molecular mass of nucleotides in DNA is 327 Da, while the average molecular mass of the eight amino acids used in this project is 132 Da. Putting these factors together, in principle, the storage density ratio of our method to the DNA method is (3/132)/(2/327) = 3.72.

About the density allowing flawless retrieval in this work, for dataset A, the total concentration of all 40 peptides was 10 ng/μL (0.25 ng/μL for each peptide) in the final mixture. Based on the injection volume of 5.0 μL, the total mass of peptides used was 10 × 5.0 = 50 ng. Therefore, the data density of peptides in this study was 848/(50 × 10^−9^) = 1.7 × 10^10^ bits/g. For dataset B, the total concentration of all 511 peptides was 1.02 μg/μL (2 ng/μL for each peptide) in the final mixture. Based on the injection volume of 5.0 μL, the total mass of peptides used was 5.1 μg. Therefore, the data density of peptides in this study was 13752/(5.1 × 10^−6^) = 2.6 × 10^9^ bits/g.

### Reporting Summary

Further information on research design is available in the Nature Research Reporting Summary linked to this article.

## Supplementary information

Supplementary Information

Peer Review File

Description of Additional Supplementary Files

Supplementary Audio 1

Reporting Summary

## Data Availability

The raw spectral data generated in this study have been deposited at MassIVE and can be available at 10.25345/C5P54Z.

## Code availability

The custom scripts for encoding data, peptide sequencing, and decoding data used in this paper are not publicly available due to the patent issues, but may be available for academic exchange and collaboration purposes by sending email requests to the corresponding authors, with the expected response time of around 1 week.
