# Supplementary material for: Data storage using peptide sequences
Source: Nat Commun. 2021 Jul 13;12:4242. doi: 10.1038/s41467-021-24496-9 (PMC8277807; doi:10.1038/s41467-021-24496-9)
Supplement: Supplementary file 1 — Supplementary Information [file 41467_2021_24496_MOESM1_ESM.pdf]

Supplementary Information

Data storage using peptide sequences

Cheuk Chi A. Ng[1,2], Wai Man Tam[3], Haidi Yin[1,2], Qian Wu[1,2], Pui-Kin So[4], Melody Yee-Man Wong[5], Francis C. M. Lau[3*], Zhong-Ping Yao[1,2*]

[1]State Key Laboratory of Chemical Biology and Drug Discovery, Research Institute for Future Food and Department of Applied Biology and Chemical Technology, The Hong Kong Polytechnic University, Hung Hom, Kowloon, Hong Kong SAR, China

[2]State Key Laboratory of Chinese Medicine and Molecular Pharmacology (Incubation) and Shenzhen Key Laboratory of Food Biological Safety Control, The Hong Kong Polytechnic University Shenzhen Research Institute, Shenzhen 518057, China

[3]Department of Electronic and Information Engineering, The Hong Kong Polytechnic University, Hung Hom, Kowloon, Hong Kong SAR, China

[4]University Research Facility in Life Sciences, The Hong Kong Polytechnic University, Hung Hom, Kowloon, Hong Kong SAR, China

[5]University Research Facility in Chemical and Environmental Analysis, The Hong Kong Polytechnic University, Hung Hom, Kowloon, Hong Kong SAR, China

*Correspondence to: Zhong-Ping Yao: zhongping.yao@polyu.edu.hk; Francis C. M. Lau: francis-cm.lau@polyu.edu.hk

Contents:

Tables S1-S5

Table S1. 40×16 block of 3-bit symbols including 850 information bits, 80 order-checking bits and 3 LDPC codes.

Symbol	S_1	S_2	S_3	S_4	S_5	S_6	S_7	S_8	S_9	S_{10}	S_{11}	S_{12}	S_{13}	S_{14}	S_{15}	S_{16}
Seq #1	$A_{1,1}$	$A_{1,2}$	$Q_{1,2}$	b_{851}	b_{852}	b_{853}	$Q_{15,16}$	$P_1^{(1)}$	$P_2^{(1)}$	$P_3^{(1)}$	$P_4^{(1)}$	$P_5^{(1)}$	$P_{201}^{(1)}$	b_{401}	b_{402}	b_{403}
			b_1	b_2	b_3	b_4	b_5	$P_1^{(2)}$	$P_2^{(2)}$	$P_3^{(2)}$	$P_4^{(2)}$	$P_5^{(2)}$	$P_{201}^{(2)}$	b_{404}	b_{405}	b_{406}
			b_6	b_7	b_8	b_9	b_{10}	$P_1^{(3)}$	$P_2^{(3)}$	$P_3^{(3)}$	$P_4^{(3)}$	$P_5^{(3)}$	$P_{201}^{(3)}$	b_{407}	b_{408}	b_{409}
Seq #2	$A_{2,1}$	$A_{2,2}$	$Q_{1,2}$	b_{854}	b_{855}	b_{856}	$Q_{15,16}$	$P_6^{(1)}$	$P_7^{(1)}$	$P_8^{(1)}$	$P_9^{(1)}$	$P_{10}^{(1)}$	$P_{202}^{(1)}$	b_{410}	b_{411}	b_{412}
			b_{11}	b_{12}	b_{13}	b_{14}	b_{15}	$P_6^{(2)}$	$P_7^{(2)}$	$P_8^{(2)}$	$P_9^{(2)}$	$P_{10}^{(2)}$	$P_{202}^{(2)}$	b_{413}	b_{414}	b_{415}
			b_{16}	b_{17}	b_{18}	b_{19}	b_{20}	$P_6^{(3)}$	$P_7^{(3)}$	$P_8^{(3)}$	$P_9^{(3)}$	$P_{10}^{(3)}$	$P_{202}^{(3)}$	b_{416}	b_{417}	b_{418}
Seq #3	$A_{3,1}$	$A_{3,2}$	$Q_{1,2}$	b_{857}	b_{858}	b_{859}	$Q_{15,16}$	$P_{11}^{(1)}$	$P_{12}^{(1)}$	$P_{13}^{(1)}$	$P_{14}^{(1)}$	$P_{15}^{(1)}$	$P_{203}^{(1)}$	b_{419}	b_{420}	b_{421}
			b_{21}	b_{22}	b_{23}	b_{24}	b_{25}	$P_{11}^{(2)}$	$P_{12}^{(2)}$	$P_{13}^{(2)}$	$P_{14}^{(2)}$	$P_{15}^{(2)}$	$P_{203}^{(2)}$	b_{422}	b_{423}	b_{424}
			b_{26}	b_{27}	b_{28}	b_{29}	b_{30}	$P_{11}^{(3)}$	$P_{12}^{(3)}$	$P_{13}^{(3)}$	$P_{14}^{(3)}$	$P_{15}^{(3)}$	$P_{203}^{(3)}$	b_{425}	b_{426}	b_{427}
⋮	⋮	⋮	⋮	⋮	⋮	⋮	⋮	⋮	⋮	⋮	⋮	⋮	⋮	⋮	⋮	⋮
Seq #10	$A_{10,1}$	$A_{10,2}$	$Q_{1,2}$	b_{878}	b_{879}	b_{880}	$Q_{15,16}$	$P_{46}^{(1)}$	$P_{47}^{(1)}$	$P_{48}^{(1)}$	$P_{49}^{(1)}$	$P_{50}^{(1)}$	$P_{210}^{(1)}$	b_{482}	b_{483}	b_{484}
			b_{91}	b_{92}	b_{93}	b_{94}	b_{95}	$P_{46}^{(2)}$	$P_{47}^{(2)}$	$P_{48}^{(2)}$	$P_{49}^{(2)}$	$P_{50}^{(2)}$	$P_{210}^{(2)}$	b_{485}	b_{486}	b_{487}
			b_{96}	b_{97}	b_{98}	b_{99}	b_{100}	$P_{46}^{(3)}$	$P_{47}^{(3)}$	$P_{48}^{(3)}$	$P_{49}^{(3)}$	$P_{50}^{(3)}$	$P_{210}^{(3)}$	b_{488}	b_{489}	b_{490}
Seq #11	$A_{11,1}$	$A_{11,2}$	$Q_{1,2}$	b_{881}	b_{882}	b_{883}	$Q_{15,16}$	$P_{51}^{(1)}$	$P_{52}^{(1)}$	$P_{53}^{(1)}$	$P_{54}^{(1)}$	$P_{55}^{(1)}$	b_{761}	b_{491}	b_{492}	b_{493}
			b_{101}	b_{102}	b_{103}	b_{104}	b_{105}	$P_{51}^{(2)}$	$P_{52}^{(2)}$	$P_{53}^{(2)}$	$P_{54}^{(2)}$	$P_{55}^{(2)}$	b_{762}	b_{494}	b_{495}	b_{496}
			b_{106}	b_{107}	b_{108}	b_{109}	b_{110}	$P_{51}^{(3)}$	$P_{52}^{(3)}$	$P_{53}^{(3)}$	$P_{54}^{(3)}$	$P_{55}^{(3)}$	b_{763}	b_{497}	b_{498}	b_{499}
⋮	⋮	⋮	⋮	⋮	⋮	⋮	⋮	⋮	⋮	⋮	⋮	⋮	⋮	⋮	⋮	⋮
Seq #39	$A_{39,1}$	$A_{39,2}$	$Q_{1,2}$	b_{965}	b_{966}	b_{967}	$Q_{15,16}$	$P_{191}^{(1)}$	$P_{192}^{(1)}$	$P_{193}^{(1)}$	$P_{194}^{(1)}$	$P_{195}^{(1)}$	b_{845}	b_{743}	b_{744}	b_{745}
			b_{381}	b_{382}	b_{383}	b_{384}	b_{385}	$P_{191}^{(2)}$	$P_{192}^{(2)}$	$P_{193}^{(2)}$	$P_{194}^{(2)}$	$P_{195}^{(2)}$	b_{846}	b_{746}	b_{747}	b_{748}
			b_{386}	b_{387}	b_{388}	b_{389}	b_{390}	$P_{191}^{(3)}$	$P_{192}^{(3)}$	$P_{193}^{(3)}$	$P_{194}^{(3)}$	$P_{195}^{(3)}$	b_{847}	b_{749}	b_{750}	b_{751}
Seq #40	$A_{40,1}$	$A_{40,2}$	$Q_{1,2}$	b_{968}	b_{969}	b_{970}	$Q_{15,16}$	$P_{196}^{(1)}$	$P_{197}^{(1)}$	$P_{198}^{(1)}$	$P_{199}^{(1)}$	$P_{200}^{(1)}$	b_{848}	b_{752}	b_{753}	b_{754}
			b_{391}	b_{392}	b_{393}	b_{394}	b_{395}	$P_{196}^{(2)}$	$P_{197}^{(2)}$	$P_{198}^{(2)}$	$P_{199}^{(2)}$	$P_{200}^{(2)}$	b_{849}	b_{755}	b_{756}	b_{757}
			b_{396}	b_{397}	b_{398}	b_{399}	b_{400}	$P_{196}^{(3)}$	$P_{197}^{(3)}$	$P_{198}^{(3)}$	$P_{199}^{(3)}$	$P_{200}^{(3)}$	b_{850}	b_{758}	b_{759}	b_{760}

Table S2. 511×16 block of 3-bit symbols including 13752 information bits, 1533 order-checking bits and 4 RS codes.

Symbol	S_1	S_2	S_3	S_4	S_5	S_6	S_7	S_8	S_9	S_{10}	S_{11}	S_{12}	S_{13}	S_{14}	S_{15}	S_{16}
Seq #1	$A_{1,1}$	$A_{1,2}$	$A_{1,3}$	$Q_{1,2}$	$p_1^{(1)}$	$p_4^{(1)}$	$p_7^{(1)}$	$p_1^{(2)}$	$p_4^{(2)}$	$p_7^{(2)}$	$p_1^{(3)}$	$p_4^{(3)}$	$p_7^{(3)}$	$p_1^{(4)}$	$p_4^{(4)}$	$p_7^{(4)}$
				$Q_{2,3}$	$p_2^{(1)}$	$p_5^{(1)}$	$p_8^{(1)}$	$p_2^{(2)}$	$p_5^{(2)}$	$p_8^{(2)}$	$p_2^{(3)}$	$p_5^{(3)}$	$p_8^{(3)}$	$p_2^{(4)}$	$p_5^{(4)}$	$p_8^{(4)}$
				$Q_{15,16}$	$p_3^{(1)}$	$p_6^{(1)}$	$p_9^{(1)}$	$p_3^{(2)}$	$p_6^{(2)}$	$p_9^{(2)}$	$p_3^{(3)}$	$p_6^{(3)}$	$p_9^{(3)}$	$p_3^{(4)}$	$p_6^{(4)}$	$p_9^{(4)}$
Seq #2	$A_{2,1}$	$A_{2,2}$	$A_{2,3}$	$Q_{1,2}$	$p_{10}^{(1)}$	$p_{13}^{(1)}$	$p_{16}^{(1)}$	$p_{10}^{(2)}$	$p_{13}^{(2)}$	$p_{16}^{(2)}$	$p_{10}^{(3)}$	$p_{13}^{(3)}$	$p_{16}^{(3)}$	$p_{10}^{(4)}$	$p_{13}^{(4)}$	$p_{16}^{(4)}$
				$Q_{2,3}$	$p_{11}^{(1)}$	$p_{14}^{(1)}$	$p_{17}^{(1)}$	$p_{11}^{(2)}$	$p_{14}^{(2)}$	$p_{17}^{(2)}$	$p_{11}^{(3)}$	$p_{14}^{(3)}$	$p_{17}^{(3)}$	$p_{11}^{(4)}$	$p_{14}^{(4)}$	$p_{17}^{(4)}$
				$Q_{15,16}$	$p_{12}^{(1)}$	$p_{15}^{(1)}$	$p_{18}^{(1)}$	$p_{12}^{(2)}$	$p_{15}^{(2)}$	$p_{18}^{(2)}$	$p_{12}^{(3)}$	$p_{15}^{(3)}$	$p_{18}^{(3)}$	$p_{12}^{(4)}$	$p_{15}^{(4)}$	$p_{18}^{(4)}$
⋮	⋮	⋮	⋮	⋮	⋮	⋮	⋮	⋮	⋮	⋮	⋮	⋮	⋮	⋮	⋮	⋮
Seq #102	$A_{102,1}$	$A_{102,2}$	$A_{102,3}$	$Q_{1,2}$	$p_{910}^{(1)}$	$p_{913}^{(1)}$	$p_{916}^{(1)}$	$p_{910}^{(2)}$	$p_{913}^{(2)}$	$p_{916}^{(2)}$	$p_{910}^{(3)}$	$p_{913}^{(3)}$	$p_{916}^{(3)}$	$p_{910}^{(4)}$	$p_{913}^{(4)}$	$p_{916}^{(4)}$
				$Q_{2,3}$	$p_{911}^{(1)}$	$p_{914}^{(1)}$	$p_{917}^{(1)}$	$p_{911}^{(2)}$	$p_{914}^{(2)}$	$p_{917}^{(2)}$	$p_{911}^{(3)}$	$p_{914}^{(3)}$	$p_{917}^{(3)}$	$p_{911}^{(4)}$	$p_{914}^{(4)}$	$p_{917}^{(4)}$
				$Q_{15,16}$	$p_{912}^{(1)}$	$p_{915}^{(1)}$	$p_{918}^{(1)}$	$p_{912}^{(2)}$	$p_{915}^{(2)}$	$p_{918}^{(2)}$	$p_{912}^{(3)}$	$p_{915}^{(3)}$	$p_{918}^{(3)}$	$p_{912}^{(4)}$	$p_{915}^{(4)}$	$p_{918}^{(4)}$
Seq #103	$A_{103,1}$	$A_{103,2}$	$A_{103,3}$	$Q_{1,2}$	b_1	b_4	b_7	b_{10}	b_{13}	b_{16}	$p_{919}^{(3)}$	$p_{922}^{(3)}$	$p_{925}^{(3)}$	$p_{919}^{(4)}$	$p_{922}^{(4)}$	$p_{925}^{(4)}$
				$Q_{2,3}$	b_2	b_5	b_8	b_{11}	b_{14}	b_{17}	$p_{920}^{(3)}$	$p_{923}^{(3)}$	$p_{926}^{(3)}$	$p_{920}^{(4)}$	$p_{923}^{(4)}$	$p_{926}^{(4)}$
				$Q_{15,16}$	b_3	b_6	b_9	b_{12}	b_{15}	b_{18}	$p_{921}^{(3)}$	$p_{924}^{(3)}$	$p_{927}^{(3)}$	$p_{921}^{(4)}$	$p_{924}^{(4)}$	$p_{927}^{(4)}$
Seq #104	$A_{104,1}$	$A_{104,2}$	$A_{104,3}$	$Q_{1,2}$	b_{19}	b_{22}	b_{25}	b_{28}	b_{31}	b_{34}	$p_{928}^{(3)}$	$p_{931}^{(3)}$	$p_{934}^{(3)}$	$p_{928}^{(4)}$	$p_{931}^{(4)}$	$p_{934}^{(4)}$
				$Q_{2,3}$	b_{20}	b_{23}	b_{26}	b_{29}	b_{32}	b_{35}	$p_{929}^{(3)}$	$p_{932}^{(3)}$	$p_{935}^{(3)}$	$p_{929}^{(4)}$	$p_{932}^{(4)}$	$p_{935}^{(4)}$
				$Q_{15,16}$	b_{21}	b_{24}	b_{27}	b_{30}	b_{33}	b_{36}	$p_{930}^{(3)}$	$p_{933}^{(3)}$	$p_{936}^{(3)}$	$p_{930}^{(4)}$	$p_{933}^{(4)}$	$p_{936}^{(4)}$
⋮	⋮	⋮	⋮	⋮	⋮	⋮	⋮	⋮	⋮	⋮	⋮	⋮	⋮	⋮	⋮	⋮
Seq #154	$A_{154,1}$	$A_{154,2}$	$A_{154,3}$	$Q_{1,2}$	b_{919}	b_{922}	b_{925}	b_{928}	b_{931}	b_{934}	$p_{1378}^{(3)}$	$p_{1381}^{(3)}$	$p_{1384}^{(3)}$	$p_{1378}^{(4)}$	$p_{1381}^{(4)}$	$p_{1384}^{(4)}$
				$Q_{2,3}$	b_{920}	b_{923}	b_{926}	b_{929}	b_{932}	b_{935}	$p_{1379}^{(3)}$	$p_{1382}^{(3)}$	$p_{1385}^{(3)}$	$p_{1379}^{(4)}$	$p_{1382}^{(4)}$	$p_{1385}^{(4)}$
				$Q_{15,16}$	b_{921}	b_{924}	b_{927}	b_{930}	b_{933}	b_{936}	$p_{1380}^{(3)}$	$p_{1383}^{(3)}$	$p_{1386}^{(3)}$	$p_{1380}^{(4)}$	$p_{1383}^{(4)}$	$p_{1386}^{(4)}$
Seq #155	$A_{155,1}$	$A_{155,2}$	$A_{155,3}$	$Q_{1,2}$	b_{937}	b_{940}	b_{943}	b_{946}	b_{949}	b_{952}	b_{7309}	b_{7312}	b_{7315}	b_{7318}	b_{7321}	b_{7324}
				$Q_{2,3}$	b_{938}	b_{941}	b_{944}	b_{947}	b_{950}	b_{953}	b_{7310}	b_{7313}	b_{7316}	b_{7319}	b_{7322}	b_{7325}
				$Q_{15,16}$	b_{939}	b_{942}	b_{945}	b_{948}	b_{951}	b_{954}	b_{7311}	b_{7314}	b_{7317}	b_{7320}	b_{7323}	b_{7326}
Seq #156	$A_{156,1}$	$A_{156,2}$	$A_{156,3}$	$Q_{1,2}$	b_{955}	b_{958}	b_{961}	b_{964}	b_{967}	b_{970}	b_{7327}	b_{7330}	b_{7333}	b_{7336}	b_{7339}	b_{7342}
				$Q_{2,3}$	b_{956}	b_{959}	b_{962}	b_{965}	b_{968}	b_{971}	b_{7328}	b_{7331}	b_{7334}	b_{7337}	b_{7340}	b_{7343}
				$Q_{15,16}$	b_{957}	b_{960}	b_{963}	b_{966}	b_{969}	b_{972}	b_{7329}	b_{7332}	b_{7335}	b_{7338}	b_{7341}	b_{7344}
⋮	⋮	⋮	⋮	⋮	⋮	⋮	⋮	⋮	⋮	⋮	⋮	⋮	⋮	⋮	⋮	⋮
Seq #485	$A_{485,1}$	$A_{485,2}$	$A_{485,3}$	$Q_{1,2}$	b_{6877}	b_{6880}	b_{6883}	b_{6886}	b_{6889}	b_{6892}	b_{13249}	b_{13252}	b_{13255}	b_{13258}	b_{13261}	0
				$Q_{2,3}$	b_{6878}	b_{6881}	b_{6884}	b_{6887}	b_{6890}	b_{6893}	b_{13250}	b_{13253}	b_{13256}	b_{13259}	b_{13262}	b_{13264}
				$Q_{15,16}$	b_{6879}	b_{6882}	b_{6885}	b_{6888}	b_{6891}	b_{6894}	b_{13251}	b_{13254}	b_{13257}	b_{13260}	b_{13263}	b_{13265}
⋮	⋮	⋮	⋮	⋮	⋮	⋮	⋮	⋮	⋮	⋮	⋮	⋮	⋮	⋮	⋮	⋮
Seq #508	$A_{508,1}$	$A_{508,2}$	$A_{508,3}$	$Q_{1,2}$	b_{7291}	b_{7294}	b_{7297}	b_{7300}	b_{7303}	b_{7306}	b_{13640}	b_{13643}	b_{13646}	b_{13649}	b_{13652}	0
				$Q_{2,3}$	b_{7292}	b_{7295}	b_{7298}	b_{7301}	b_{7304}	b_{7307}	b_{13641}	b_{13644}	b_{13647}	b_{13650}	b_{13653}	b_{13655}
				$Q_{15,16}$	b_{7293}	b_{7296}	b_{7299}	b_{7302}	b_{7305}	b_{7308}	b_{13642}	b_{13645}	b_{13648}	b_{13651}	b_{13654}	b_{13656}
Seq #509	$A_{509,1}$	$A_{509,2}$	$A_{509,3}$	$Q_{1,2}$	b'_{1}	b'_{4}	b'_{7}	b'_{10}	b'_{13}	b'_{16}	b'_{55}	b'_{58}	b'_{61}	b'_{64}	0	0
				$Q_{2,3}$	b'_{2}	b'_{5}	b'_{8}	b'_{11}	b'_{14}	b'_{17}	b'_{56}	b'_{59}	b'_{62}	b'_{65}	b'_{67}	0
				$Q_{15,16}$	b'_{3}	b'_{6}	b'_{9}	b'_{12}	b'_{15}	b'_{18}	b'_{57}	b'_{60}	b'_{63}	b'_{66}	b'_{68}	0
Seq #510	$A_{510,1}$	$A_{510,2}$	$A_{510,3}$	$Q_{1,2}$	b'_{19}	b'_{22}	b'_{25}	b'_{28}	b'_{31}	b'_{34}	b'_{69}	b'_{72}	b'_{75}	b'_{78}	0	0
				$Q_{2,3}$	b'_{20}	b'_{23}	b'_{26}	b'_{29}	b'_{32}	b'_{35}	b'_{70}	b'_{73}	b'_{76}	b'_{79}	b'_{81}	0
				$Q_{15,16}$	b'_{21}	b'_{24}	b'_{27}	b'_{30}	b'_{33}	b'_{36}	b'_{71}	b'_{74}	b'_{77}	b'_{80}	b'_{82}	0
Seq #511	$A_{511,1}$	$A_{511,2}$	$A_{511,3}$	$Q_{1,2}$	b'_{37}	b'_{40}	b'_{43}	b'_{46}	b'_{49}	b'_{52}	b'_{83}	b'_{86}	b'_{89}	b'_{92}	0	0
				$Q_{2,3}$	b'_{38}	b'_{41}	b'_{44}	b'_{47}	b'_{50}	b'_{53}	b'_{84}	b'_{87}	b'_{90}	b'_{93}	b'_{95}	0
				$Q_{15,16}$	b'_{39}	b'_{42}	b'_{45}	b'_{48}	b'_{51}	b'_{54}	b'_{85}	b'_{88}	b'_{91}	b'_{94}	b'_{96}	0

Table S3. Description of the peptide sequencing problem.

	Peptide sequencing problem
Input	• set *A* of amino acids;<br>• mass set for the amino acids in set *A*;<br>• experimental spectrum *S;*<br>• set (*m/z*) of mass/charge ratio of spectrum *S*;<br>• set *I* of intensity of spectrum *S*;<br>• length *N* of the peptide sequence;<br>• fixed C- and N- terminal masses of Peptide *P*;
Output	• mass M of the whole sequence.<br>• Peptide P of length N.
Problem	• find peptide P, which most likely generates the experimental spectrum S.

Table S4. The 40 peptides encoding dataset A.

Peptide#	Sequence	Peptide#	Sequence
1	FSSFSYTLTFLASAEAER	21	FEASEYYEFYTATEVAFR
2	FSTYYETFSSLVYVLATR	22	FEVYSTSSVVFAVFSELR
3	FSETYSSYLYELATLSYR	23	FELSYYSFVFFTETALSR
4	FSYTSETLSYSAVETSSR	24	FEFYTTEYAAETVTTEVR
5	FSASYEYEAYEFELVSYR	25	FYSLTETLSESVFLTLLR
6	FSVESTYVLSYTVEAFFR	26	FYTFTETLLAAELYFATR
7	FSLSYSEFTFETESLYTR	27	FYEAYETLFLTASYLFTR
8	FSFSTSYLFYFESFLSSR	28	FYYVSESELTSVFSTYVR
9	FTSFTEYFLSLYLVTFSR	29	FYAEESTEASTTSETTLR
10	FTTVTSYLFFFYSTFLYR	30	FYVYSTSATAFYVFSFLR
11	FTETSSESFASTLYLYVR	31	FYLTSSELSEVAYTEFER
12	FTYSEYSESFATVYEYAR	32	FYFTTSEYVAAYETEEFR
13	FTASYTTELVAVLASTER	33	FASFTEYYLTASLFSELR
14	FTVESTEVLALFELSYYR	34	FATVTSYLFTELVTSASR
15	FTLSTSYLVYVYEAFFSR	35	FAEVEYESATALYTAAVR
16	FTFTESESLAYVAVEEFR	36	FAYAEYSFSAELSYVTTR
17	FESFTESTATEELLEALR	37	FAALETTAFEALEATAYR
18	FETLYEYLYVTTFLVLTR	38	FAVYSTSSYFVYYSSSER
19	FEEFEYSFYFTYVYAYYR	39	FALSSEYVTAASTSEESR
20	FEYTYYSLFEAFATTVSR	40	FAFSYSTSFELFLYFTLR

Table S5. (a) The number of peptides with 0-16 correct amino acids after sequencing and (b) number of error symbols found for dataset B.

(a)

No. of correct amino acids	No. of peptides
0	5
1	0
2	0
3	2
4	6
5	8
6	6
7	4
8	6
9	1
10	2
11	1
12	2
13	7
14	27
15	0
16	434
Correct amino acids	7659

(b)

	No. of error symbols	Maximum allowed
RS1	40	51
RS2	43	51
RS3	55	77
RS4	67	77
