# Supplementary material for: Data storage using peptide sequences
Source: Nat Commun. 2021 Jul 13;12:4242. doi: 10.1038/s41467-021-24496-9 (PMC8277807; doi:10.1038/s41467-021-24496-9)
Supplement: Supplementary file 3 — Description of Additional Supplementary Files [file 41467_2021_24496_MOESM3_ESM.pdf]

Description of Additional Supplementary Files

File Name: Supplementary Audio 1
Description: The music “Silent Night”
