# Supplementary material for: Data storage using peptide sequences
Source: Nat Commun. 2021 Jul 13;12:4242. doi: 10.1038/s41467-021-24496-9 (PMC8277807; doi:10.1038/s41467-021-24496-9)
Supplement: Supplementary file 2 — Peer Review File [file 41467_2021_24496_MOESM2_ESM.pdf]

Editorial Note: This manuscript has been previously reviewed at another journal that is not operating a transparent peer review scheme. The manuscript was considered suitable for publication without further review at Nature Communications.
